# Tandem Dye-Doped Nanoparticles for NIR Imaging via Cerenkov Resonance Energy Transfer

**DOI:** 10.3389/fchem.2020.00071

**Published:** 2020-02-27

**Authors:** Damiano Genovese, Luca Petrizza, Luca Prodi, Enrico Rampazzo, Francesco De Sanctis, Antonello Enrico Spinelli, Federico Boschi, Nelsi Zaccheroni

**Affiliations:** ^1^Department of Chemistry “Giacomo Ciamician”, University of Bologna, Bologna, Italy; ^2^Immunologic Section, Department of Medicine, Policlinico G.B. Rossi, Verona, Italy; ^3^Experimental Imaging Centre, San Raffaele Scientific Institute, Milan, Italy; ^4^Department of Computer Science, University of Verona, Verona, Italy

**Keywords:** luminescence, Cerenkow radiation, near-infrared region imaging, silica nanoparticles, energy transfer, radioisotopes

## Abstract

The detection of the Cerenkov radiation (CR) is an emerging preclinical imaging technique which allows monitoring the *in vivo* distribution of radionuclides. Among its possible advantages, the most interesting is the simplicity and cost of the required instrumentation compared, e.g., to that required for PET scans. On the other hand, one of its main drawbacks is related to the fact that CR, presenting the most intense component in the UV-vis region, has a very low penetration in biological tissues. To address this issue, we present here multifluorophoric silica nanoparticles properly designed to efficiently absorb the CR radiation and to have a quite high fluorescence quantum yield (0.12) at 826 nm. Thanks to a highly efficient series of energy transfer processes, each nanoparticle can convert part of the CR into NIR light, increasing its detection even under 1.0-cm thickness of muscle.

## Introduction

Cerenkov luminescence imaging (CLI) is a novel preclinical modality to image the biodistribution of radiotracers, based on the detection of Cerenkov radiation (CR) using optical imaging (Spinelli et al., 2010). CR is generated by charged particles, generally electrons (β−) and positrons (β+), traveling faster than the speed of light in a dielectric medium, which, in the case of biomedical applications, are water and tissues (Spinelli and Boschi, 2015). According to the Frank–Tamm theory (Jelley, 1958), the CR originated by different particles presenting the same spectral shape—that in water is UV weighted being inversely proportional to the squared wavelength—but with different intensities that are dependent on the beta endpoint energy. It is thus possible to enable a broad range of applications, taking profit of CR escaping from living subjects, to monitor the biodistribution of radioisotopes by optical imaging as an alternative to PET (Yang et al., 2015). The use of CR for bioimaging is, however, limited by a very low penetration in biological tissues—relatively transparent only in the region of 650–800 nm—since it presents its most intense component in the UV-vis region. Therefore, a shift in the CR spectrum in the near-infrared region (NIR) would improve the detection of radionuclides deeper inside the tissues and increase the output signal. This shift can be obtained through the so-called Cerenkov resonance energy transfer (CRET) process (Hu et al., 2014) that has been also used to sensitize porphyrins in photodynamic therapy (Ni et al., 2018). Considering the broad wavelength range of CR, the use of a single organic dye acceptor might limit the performance of CRET; for this reason, Bernhard et al. have developed a methodology based on a mixture of fluorophores having a good spectral overlap to obtain a nearly two-fold radiance increase compared to a tumor injected with just the radionuclide (Bernhard et al., 2017). However, an energy transfer cascade can be hardly optimized for freely diffusing dyes (Shaffer et al., 2017) but can be precisely engineered using nanoparticles that could, therefore, represent a valuable alternative.

Early works showing a CR wavelength shift (e.g., red light signal increase) took advantage of quantum dots (QDs) (Dothager et al., 2010; Liu et al., 2010; Boschi and Spinelli, 2012; Thorek et al., 2013; Hu et al., 2014) that show a very high Stokes shift. However, containing toxic elements like cadmium, they not only require a careful external passivation to be used in *in vivo* imaging experiments but also give rise severe concerns related to their preparation and disposal procedures. Alternative materials would, therefore, be of interest for this kind of applications, when characterized by minimal safety and environmental risks throughout their whole lifetime, and different radiotracers and nanoparticles were recently reviewed in literature (Shaffer et al., 2017). Among all them, we believe that dye-doped silica nanoparticles (DDSNs) represent the alternative of choice (Bonacchi et al., 2011; Montalti et al., 2014; Ma et al., 2015; Zhang et al., 2016; Rampazzo et al., 2018), especially considering that the benign nature of the material is accompanied by the possibility to tune their photophysical properties. In this scenario, by exploiting the organization of different kinds of dyes within Pluronic F127 silica nanoparticles (PluS NPs), we had already obtained large shifts of blue light excitation into NIR emission through a very efficient energy transfer cascade, overcoming all problems related to freely diffusing dyes. PluS NPs can be obtained through a versatile surfactant-aided strategy in very mild conditions, using Pluronic F127 micelles as templates. This nanomaterial displays the typical advantages of both silica and PEG, such as water solubility and non-toxicity, is very stable in water, also from a photochemical point of view, with reproducible size and properties (silica core of 10-nm diameter and an outer PEG shell resulting in an overall hydrodynamic diameter of 25 nm). In addition, these nanostructures can be tailored with properties suitable to a specific application and equipment.

## Materials and Methods

### Chemicals

The 1,1,2-trimethylbenz[e]indole (≥98%), 6-iodo-1-hexyne (97%), acetonitrile (99.8%), diethyl ether (≥99.8%), acetic anhydride (≥99%), malonaldehyde bis(phenylimine) monohydrochloride (97%), dichloromethane (≥99.8%), methanol (≥99.8%), pyridine (≥99.8%), acetic acid (≥99.5%), tert-butyl 3-(azidomethyl)piperidine-1-carboxylate (CPR), sodium ascorbate (≥99%), copper(II) sulfate pentahydrate (≥98%), lithium chloride (≥99%), sodium sulfate (≥99%), chloroform (≥99.8%), chloroform-d (99.8 atom % D), methanol-d4 (99.8 atom % D), dimethyl sulfoxide-d6 (DMSO-d6; 99.8 atom % D), 1-[bis(dimethylamino)methylene]-1H-1,2,3-triazolo[4,5-b]pyridinium-3-oxid-hexafluoro phosphate (97%), 4-mercaptobenzoic acid (99%), IR-775 chloride (dye content ~90%) (3-aminopropyl)triethoxysilane (APTES, 99%), N,N-diisopropylethylamine (DIPEA, ≥99%) and N,N-dimethylformamide (DMF, ≥99.8 %) and Silica on TLC Alu foils (4 × 8 cm, with fluorescent indicator of 254 nm) were purchased from Sigma-Aldrich.

### Experimental Procedures

PluS NPs and fluorescent dyes **CU**, **BO**, and **RB** were prepared as previously reported (Rampazzo et al., 2014). Experimental details for the synthesis of cyanine dyes **C5** and **C7** are reported in the Supplementary Information file.

### Optical Imaging Acquisitions and Simulations

Optical images were acquired using the IVIS Spectrum optical imager (Perkin Elmer, Massachusetts, USA). The IVIS Spectrum is based on a cooled (−90°C) back-thinned, back-illuminated CCD camera. The CCD has an active array of 1,920 × 1,920 pixels with a size equal to 13 × 13 μm. Images calibrated in radiance units (p/s/cm^2^/sr) were corrected for dark measurements and cosmic rays. The efficiency, total flux, and average radiance were measured by drawing regions of interest (ROIs) over the images. Image acquisition, processing, and analysis were performed with Living Image 4.5 (Perkin Elmer).

Fluorescent images of PluS NPs alone (300 μl in non-fluorescent 96-multiwell plate) were acquired in fluorescence modality using all the excitation filters (430, 465, 500, 535, 570, 605, 640, 675, 710, and 745 nm) and emission filters (500, 520, 540, 560, 600, 620, 640, 660, 680, 700, 720, 740, 760, 780, 800, 820, and 840 nm) mounted on the IVIS instrument. Avoiding possible overlapping between excitation and emission filters, 111 combinations of excitation emission filters were tested [exposure time = 1 s, f/2, binning (B) = 8 and field of view (FoV) = 13 cm].

Optical images of CR (32P-ATP, 300 μl in non-fluorescent 96-multiwell plate) were acquired in bioluminescence modality (without excitation light and with different emission filters or without emission filters) with exposure time = 300 s, f/2, B = 8, and FoV = 13 cm. With the last modality and the same exposure parameters, optical images of CR + PluS NPs were acquired.

Since induced fluorescence is related the beta emission of the radioisotope more than the labeled molecule, ^32^P-ATP was used in the experiments. ^32^P has been used in the clinic for the treatment of bone metastasis (Gordon Smart, 1965), polycythemia vera (Najean and Rain, 1997), and thrombocythemia (Shetty-Alva and Cheng, 2006). Moreover, the ^32^P-ATP was found to show antitumoral activity in preclinical studies (Galiè et al., 2017).

When monochromatic photons travel in a slab of material, they can be absorbed; the relationship between the intensity of the transmitted light (*I*_1_) and the intensity of the incident light (*I*_0_) is expressed by the Lambert–Beer law.

(1) $$\begin{array}{l} I_{1}=\;{I_{0}e}^{-\mu x} \end{array}$$

where μ is the attenuation (absorption, cm^−1^) coefficient, and *x* (cm) is the thickness of the slab. Applying the Lambert–Beer law to the CR+ PluS NP spectrum measured experimentally and considering the optical property of the muscle tissue, which is the most used tissue for optical simulations, a computational evaluation of the CR+ PluS NP light emission escaping from the muscle slab can be made. In particular, regarding the optical properties of muscle tissue, the absorption coefficient was considered and its values extracted from the Living Image software database (for example for the minimum and maximum wavelengths explored in this study, μ = 2.764 at 500 nm and μ = 0.594 at 840 nm). CR alone and CR+ PluS NP sources located at 0.5- and 1.0-cm depth in the muscle were considered in order to investigate the emission at the surface due to an intermediate and deeper source in the tissue, which could mimic real sources in *in vivo* preclinical studies.

### Photophysical Measurements

UV-VIS absorption spectra were recorded at 25°C by means of a Perkin-Elmer Lambda 45 spectrophotometer. Quartz cuvettes with an optical path length of cm were used. The fluorescence spectra were recorded with an Edinburgh FLS920-equipped photomultiplier Hamamatsu R928P for emissions below 800 nm and a low-noise Edinburgh Instruments Germanium detector with a dedicated grating for emissions in the range of 800–1,400 nm. Luminescence quantum yields (Φ, uncertainty ± 15%) were determined using solutions of Rhodamine 101 in methanol (Φ = 1.00), Quinine sulfate in H_2_SO_4_ (0.05 M; Φ = 0.53), Fluorescein in NaOH (0.1 M; Φ = 0.92) (Brouwer, 2011), and IR 125 in DMSO (Φ = 0.13) (Rurack and Spieles, 2011). Fluorescence intensities were corrected for inner filter effects according to standard methods (Credi and Prodi, 2014).

## Results and Discussion

### Photophysical Properties of the Nanoparticles

**Scheme 1:**
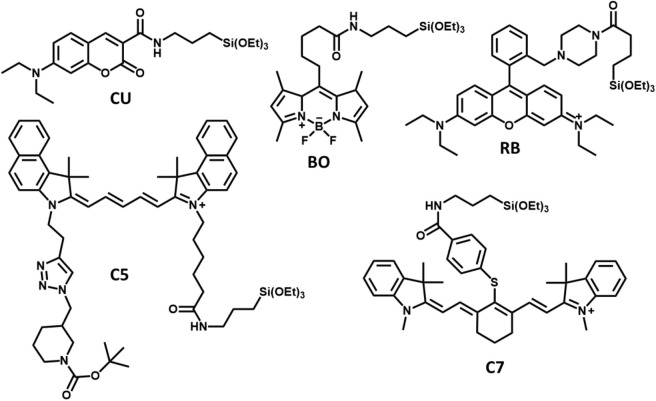
Molecular structures of the triethoxysilane functionalized dyes used to dope the pluronic–silica nanoparticles (PluS NPs) reported in this study.

We synthesized pluronic–silica (PluS) nanoparticles doped with five different dyes that have been chosen to efficiently absorb CR in all the visible ranges and to efficiently funnel the excitation energy toward the lowest energy dye, a Cy7 derivative, presenting a fluorescence emission in the near-infrared region (NIR). The molecular structures of the different doping units are shown in Scheme 1, and they all present a triethoxysilane moiety for the covalent bonding to the silica matrix of the nanoparticle. The triethoxysilane derivatives of DEAC (**CU**), tetramethyl bodipy (**BO**), Cy5.5 (**C5**), and Cy7 (**C7**) were obtained by amide coupling with 3-aminopropyltriethoxy silane, while the rhodamine B derivative (**RB**) was obtained by reaction of the corresponding rhodamine B piperazine amide derivative with 3-isocyanatopropyltriethoxysilane.

**Figure 1:**
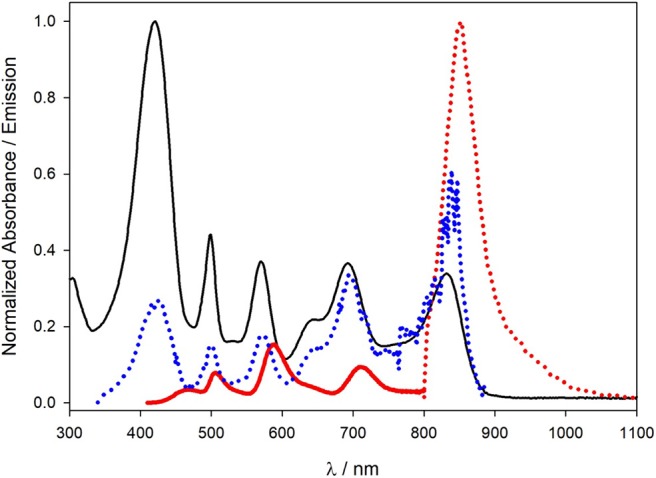
Absorption (black line), fluorescence (λexc = 422 nm; red full line in the 400–800-nm range, red dotted line 800–1,100-nm range), and excitation (λem = 826 nm; blue dotted line) spectra of a water dispersion of nanoparticles (NPs).

To maximize the absorption of the CR radiation and to optimize the antenna effect toward the lower-energy dye, we have chosen to dope the NPs with a large number of CU (% doping 0.50) and of C7 units (% doping 0.40), respectively. The average number of dyes effectively inserted inside each NP is summarized in Table 1 and well correlates with the doping percentages used during the synthesis. As can be seen in Table 1 and Figure 1, the resulting NPs present very high molar absorption coefficients in different regions of the visible spectrum, so that CR is expected to be efficiently collected. All dyes are also highly quenched [more than 10-fold with respect to the free dye in solution (Rampazzo et al., 2014)] except for C7 that has a quantum yield very similar to the one observed for the free dye in methanol. In particular, upon excitation at 420 nm, where the absorption is dominated by the band of CU, the fluorescence spectrum (Figure 1, red lines) shows a quite weak band at 462 nm, three other bands with comparable intensities at 505, 588, and 709 nm, and, finally, a quite strong band at 851 nm. A similar behavior (spectra not shown) has been observed upon excitation at 499 nm (BO band), at 569 nm (RB band), and at 700 nm (C5 band). For the sake of precision, the fluorescence spectrum reported in Figure 1 has been obtained with two different detectors (phototube in the 400–800-nm region and a Ge detector in the 800–1,100-nm), and although suitable corrections have been made, some distortions, especially around 800 nm, could be present. All these data show that, upon excitation of a given dye, there is a series of energy transfer processes to the dyes having lower-energy excited states, leading to the generation of the excited state of C7—responsible for the intense fluorescence at 851 nm—with high efficiency. The excitation spectrum recorded at λem = 900 nm (Figure 1) further supports this interpretation. As can be seen, this spectrum reproduces the profile of the absorption spectrum showing the same bands, indicating that, wherever the excitation is performed, the energy transfer processes among the fluorophores efficiently funnel the excitation energy to the C7 moieties. The band of CU and, to a lesser extent, of BO are less intense in the normalized excitation spectrum with respect to what is observable in the absorption one, a finding that can be explained considering that even if each energy transfer step has a high efficiency, a noticeable loss can be observed if many steps are necessary to populate the lowest energy dye.

**Table 1:** Dye content and photophysical properties of NPs.

**Dye**	**% Doping^a^**	**N° dyes/NP**	**λ_**max**_ abs (nm)**	**ε (M^**−1**^ cm^**−1**^)**	**λ_**max**_ em (nm)**	**Φ (dye@NPs)**
CU	0.50	27	422	1,186,000	462	0.002
BO	0.15	19	499	881,000	505	0.02
RB	0.15	10	569	983,000	588	0.05
C5	0.25	10	700	1,458,000	709	0.01
C7	0.40	17	832	2,542,000	851	0.12

a *(mol dye/mol TEOS) × 100*.

### Optical Imaging Acquisitions

**Figure 2:**
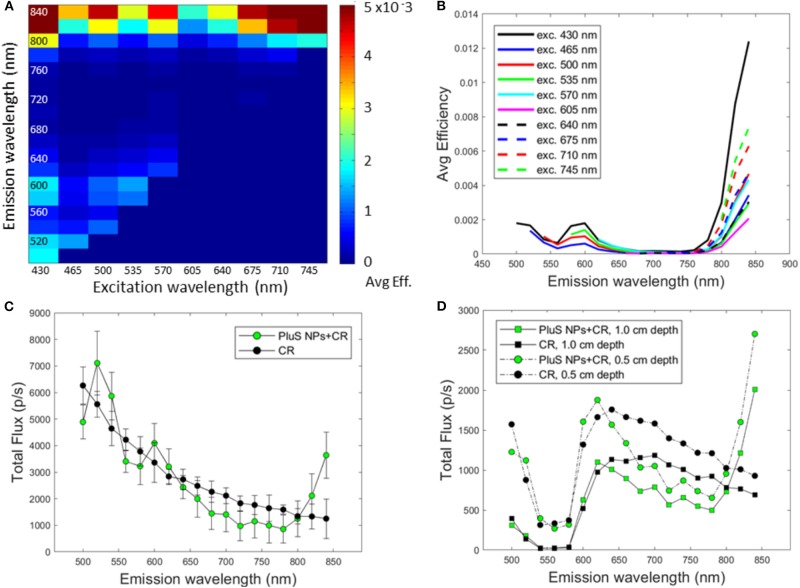
NP optical properties. Excitation and emission map **(A)**, emission spectra obtained with different excitation wavelengths **(B)**, CR-stimulated emission spectrum in comparison with pure CR spectrum **(C)**, computed spectra of CR-stimulated NPs located 0.5 and 1 cm below the surface of a slab of biological tissue (muscle) **(D)**.

The excitation/emission map recorded during optical imaging experiments is shown in Figure 2A. All the investigated excitation wavelengths gave rise to a high fluorescence emission in the near-infrared range (NIR) range (800–840) nm; in fact, with all the filters (430, 675, 710, and 745 nm), the efficiency measured in the NIR by the IVIS spectrum was >4.5 10^−3^.

The emission spectra obtained with different excitation wavelengths (430, 465, 500, 535, 570, 605, 640, 675, 710, and 745 nm) are presented in Figure 2B. The results related to the last three excitation wavelengths are particularly promising for *in vivo* imaging applications that require, indeed, together with a high efficiency, both excitation and emission wavelengths inside the optical tissue window (650–850 nm). It has to be mentioned that, also in these conditions, a residual emission from higher-energy dyes can be observed.

The CR spectrum of ^32^P-ATP measured in the 500–840-nm range, normalized at 720 nm where the PluS NPs show the lowest average efficiency, is reported in Figure 2C. The light intensity rapidly decreases increasing the emission wavelength, and the Cerenkov emission is highly reduced with respect to 500 nm in the tissue transparency optical window.

As can be seen in the same figure (green dots), comparing this result with the CR-stimulated PluS NP emission (again normalized at 720 nm), it is possible to observe a quite different profile in the 500- to 840-nm range, since the latter one clearly results from the sum of CR radiation and the PluS NP fluorescence emission. The intensity generally decreases, increasing the excitation wavelengths, according to the CR radiation, but in three emission bands at 520–540, 600–620, and 820–840 nm (NIR band), the CR + PluS NP emission is higher than that of the CR alone. The three emission bands are, therefore, due to the CR excitation of the PluS NPs according to the emission properties shown in Figure 2A. In particular, at 840 nm, the increase with respect to the pure Cerenkov emission is 2.9-fold, that is, a higher increase respect to the one (two-fold) previously obtained with a mixture of dyes (Bernhard et al., 2017).

In order to evaluate the possible influence of the interactions among light and biological tissues, we performed a simulation to obtain the spectral features of the CR transmitted through slabs of muscle of two different thicknesses (0.5 or 1.0 cm), selected as a biological model of the absorber material. In particular, we applied the Lambert–Beer law to the spectra reported in Figure 2C of the CR radiation in the absence (black dots) and presence of the PluS NPs (green dots), considering the optical properties of the absorber, as reported in the Material and Methods section.

The simulated spectra are reported in Figure 2D. As expected, due to its very low transparency at these wavelengths, the muscle leads to a strong reduction in the signal in the 480- to 580-nm range both in the absence and presence of the PluS NPs. In particular, the hemoglobin absorption band, especially in the 540–580-nm range is clearly visible. Considering the three wavelength bands in which the CR + PluS NPs have a higher emission than CR alone, it is noticeable that the first two bands (520–540 and 620–640) are still affected by the hemoglobin absorption, so they are less useful for the *in vivo* imaging purpose. Instead, the NIR band (820–840 nm) shows a high light emission and, in particular, at 840 nm is registered the highest emission on the entire wavelength region explored. In this context, the almost three-fold increase at 840 with respect to the emission of pure CR is of particular interest. This clearly indicates that these PluS NPs could be conveniently applied to increase the performances of Cerenkov luminescence imaging in the NIR wavelength region.

## Conclusions

We have presented PluS NPs doped with five different dyes, possessing high colloidal stability and very interesting photophysical properties. In particular, these PluS NPs are characterized by a high absorption all over the visible region (with ε > 1,000,000 M^−1^ cm^−1^ at some wavelengths) and a convenient NIR emission. The excitation of the lowest energy dye (**Cy7**), that presents a relatively high fluorescence quantum yield in the NIR (Φ = 0.12) can be, in fact, obtained via very efficient energy transfer processes from the other dyes. These features, combined with the lack of toxicity demonstrated so far by this family of NPs, make them suitable for increasing the performance of Cerenkov luminescence imaging (CLI)—a technique that is receiving increasing attention for preclinical studies. Each NP, being doped with five different dyes, is able to convert part of CR into NIR fluorescence, thus improving tissue penetration—as demonstrated simulating a muscle tissue over 1 cm of thickness—overcoming the most stringent limitation of CLI. In particular, a three-time increase in the intensity at 840 nm—compared to the CR alone—has been evidenced. Noteworthy, the brightness of these NPs is very high with many different excitation/emission filter setups. This feature, together with the large excitation wavelength region, makes them suitable for imaging applications even when the available instrumentation is equipped with lasers that allow only a limited number of excitation wavelengths. These results are very encouraging to continue the research derivatizing these PluS NPs with suitable radiotracers, to test their performances *in vivo*.

## Data Availability Statement

Experimental procedures and characterizations of compound C5 and C7 are included in the article/Supplementary Material.

## Author Contributions

LPr, ER, NZ, and FB designed and supervised the project. LPe and ER prepared the dye-doped nanoparticles. DG, LPe, and ER performed the photophysical experiments. FB, FD, and AS performed the Cerenkov radiation experiments and analyzed the results. LPr, ER, NZ, DG, and FB analyzed the results and wrote the paper. LPr provided the resources related to the project. All authors reviewed the manuscript.

### Conflict of Interest

The authors declare that the research was conducted in the absence of any commercial or financial relationships that could be construed as a potential conflict of interest.
