# Supplementary material for: Tandem Dye-Doped Nanoparticles for NIR Imaging via Cerenkov Resonance Energy Transfer
Source: Front Chem. 2020 Feb 27;8:71. doi: 10.3389/fchem.2020.00071 (PMC7056810; doi:10.3389/fchem.2020.00071)
Supplement: Supplementary file 1 [file Data_Sheet_1.pdf]

Supplementary Material

1 Chemicals

The 1,1,2-trimethylbenz[e]indole (≥ 98 %), 6-iodo-1-hexyne (97 %), acetonitrile (99.8 %), diethyl ether (≥ 99.8 %), acetic anhydride (≥ 99 %), malonaldehyde bis(phenylimine) monohydrochloride (97 %), dichloromethane (≥ 99.8 %), methanol (≥ 99.8 %), pyridine (≥ 99.8 %), acetic acid (≥ 99.5 %), tert-butyl 3-(azidomethyl)piperidine-1-carboxylate (CPR), sodium ascorbate (≥ 99 %), copper(II) sulfate pentahydrate (≥ 98 %), lithium chloride (≥ 99 %), sodium sulfate (≥ 99 %), chloroform (≥ 99.8 %), chloroform-d (99.8 atom % D), methanol-d_4 (99.8 atom % D), dimethyl sulfoxide-d_6 (DMSO-d_6; 99.8 atom % D), 1-[bis(dimethylamino)methylene]-1H-1,2,3-triazolo[4,5-b]pyridinium-3-oxid-hexafluoro phosphate (97 %), 4-mercaptobenzoic acid (99 %), IR-775 chloride (dye content ~ 90 %),

2 Supplementary data

3-(hex-5-yn-1-yl)-1,1,2-trimethyl-1*H*-benzo[*e*]indol-3-ium iodide (**3**) was synthesized adapting a reported procedure.[1]

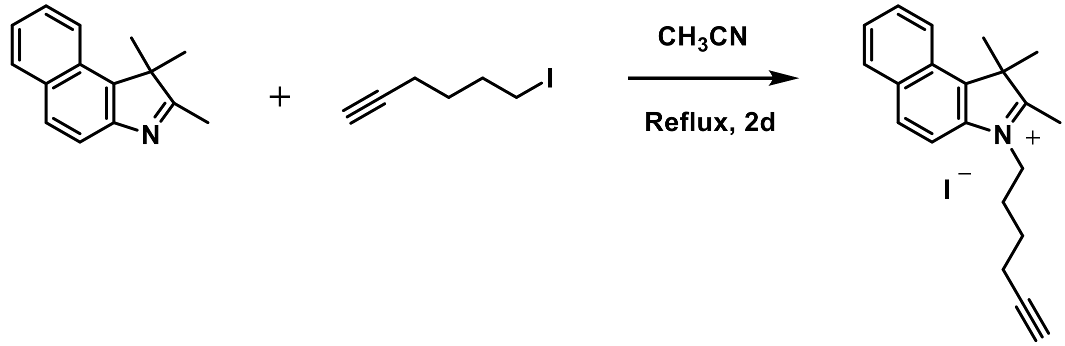

1 **2** **3**

Supplementary Figure 1: Synthesis of compound **3**.

In a 25 mL round bottom flask dried with a heat gun under a flow of Argon, to a solution of 1,1,2-Trimethylbenz[e]indol (**1**, 0.157 g, 0.8 mmol) in acetonitrile (CH_3CN, 8 mL) was added 1-Iodo-1-hexyne (**2**, 158 µL, 1.2 mmol) and the reaction mixture was heated under reflux for two days. After cooling down to room temperature, the reaction mixture was concentrated in vacuo and the resulting compound was washed three times with Diethyl Ether to give the compound **3** as dark green solid (0.208 g, yield 90 %).

^{1}H-NMR (400 MHz, $CDCl_3$, 25°C) δ = 8.10 (2H, t, *J* = 9.4 Hz, ar. C*H*), 8.05 (1H, d, *J* = 8.4 Hz, ar. C*H*), 7.88 (1H, d, *J* = 8.8 Hz, ar. C*H*), 7.74 (1H, t, *J* = 7.6 Hz, ar. C*H*), 7.67 (1H, t, *J* = 7.6 Hz, ar. C*H*), 4.86 (2H, t, *J* = 7.6 Hz, -NCH_2-), 3.23 (3H, s, -NCCH_3), 2.37-2.33 (2H, m, CHCCH_2-), 2.21-2.13 (2H, m, -NCH_2CH_2-), 1.99-1.98 (1H, m, -CC*H*), 1.88 (6H, s, -CH_3), 1.82-1.75 (2H, m, -NCH_2 CH_2CH_2-).

^{13}C-NMR (100 MHz, $CDCl_3$, 25°C) δ = 194. 8, 153.3, 137.4, 136.3, 132.9, 130.8, 129.3, 128.0, 126.9, 122.2, 112.1, 82.4, 69.4, 55.2, 48.8, 26.2, 24.5, 22.0, 17.1, 16.2.

ESI-MS m/z calculated for $C_{21}H_{24}N^+$ = 290.2; obs. = 290.2.

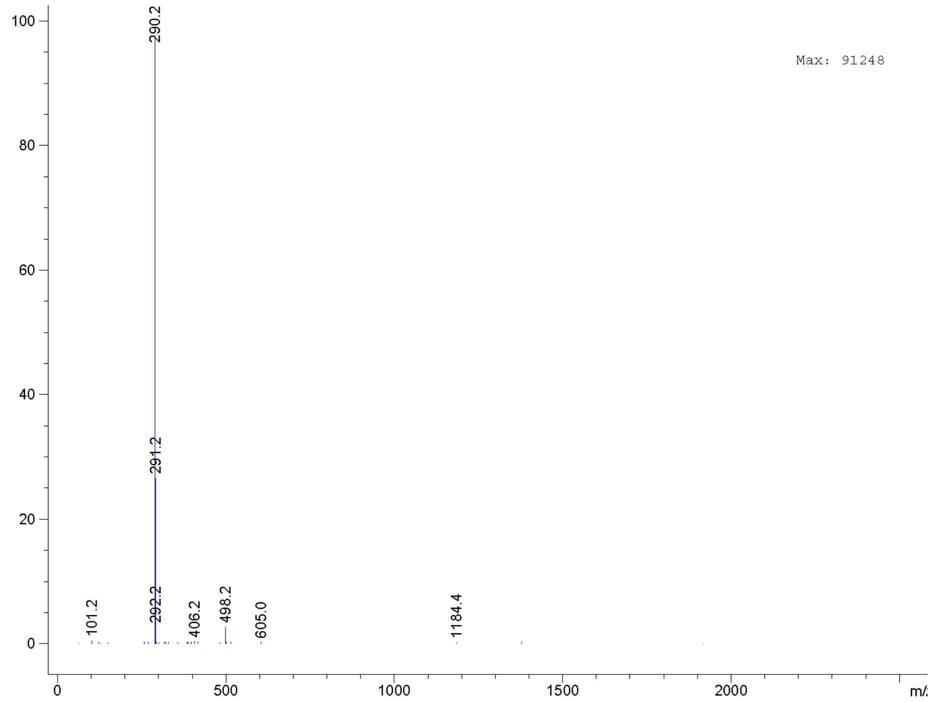

Supplementary Figure 2: ESI-MS spectra of compound **3**.

3-(hex-5-yn-1-yl)-1,1-dimethyl-2-((1*E*,3*E*)-4-(*N*-phenylacetamido)buta-1,3-dien-1-yl)-1*H*-benzo[*e*]indol-3-ium iodide (**5**) was synthesized adapting a reported procedure.[1]

.

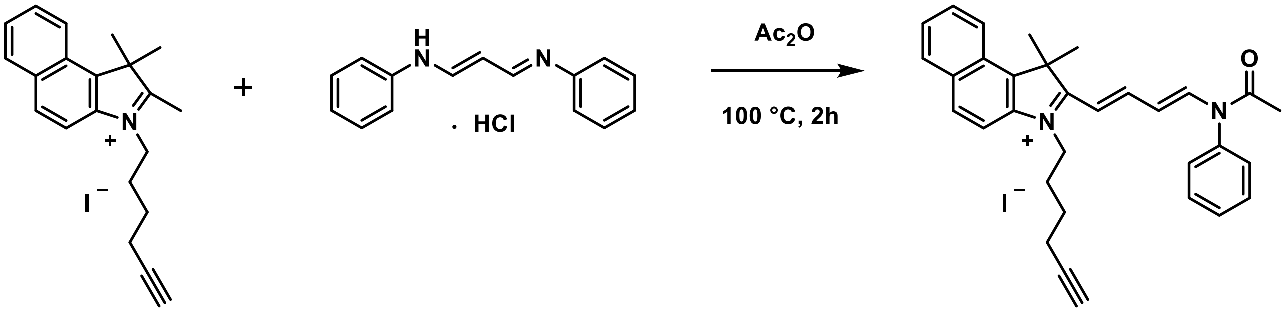

3 **4** **5**

Supplementary Figure 3: Synthesis of compound **5**.

In a 25 mL round bottom flask dried with a heat gun under a flow of argon, a suspension of the compound **3** (0.208 g, 0.7 mmol) and malonaldehyde bis(phenylimine) monohydrochloride (**4**, 0.181

g, 0.7 mmol) in acetic anhydride (Ac_2O, 7 mL) was heated at 100 °C for 2h. After cooling down to room temperature, the reaction mixture was poured into 250 mL of diethyl ether. The brown precipitate was then filtered (**5**, 0.218 g, yield 67 %) and used in the next step without further purification. R_f = 0.46 (silica, dichloromethane/methanol = 90/10).

ESI-MS m/z calculated for $C_{32}H_{33}N_2O^+$ = 461.3; obs. = 461.2.

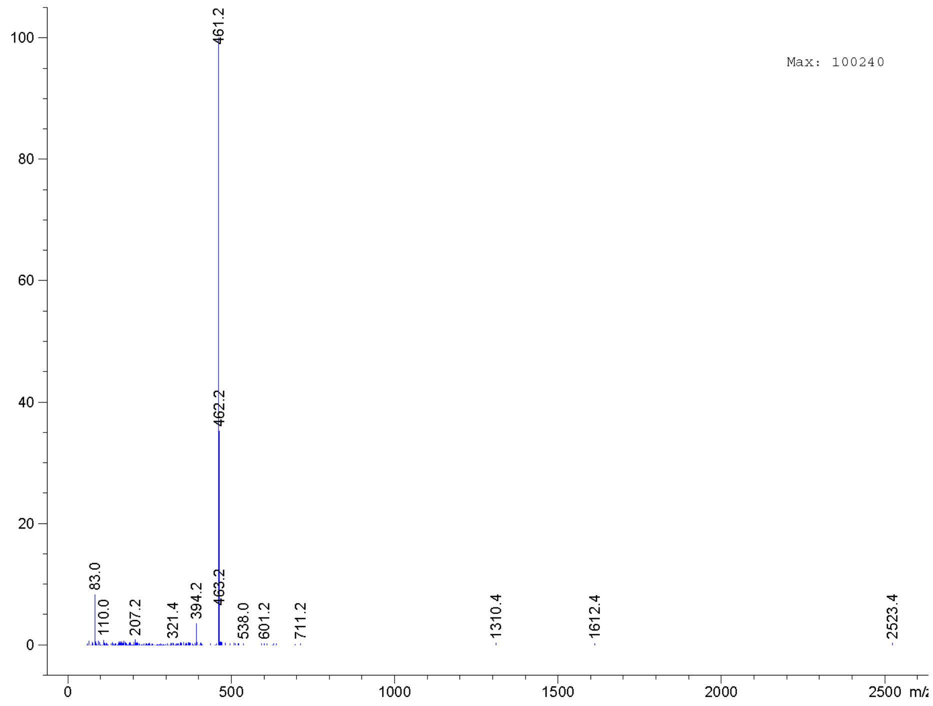

Supplementary Figure 4: ESI-MS spectra of compound **5**.

6-(1,1,2-trimethyl-1H-benzo[e]indol-3-ium-3-yl)hexanoate (**6**) was synthesized following a reported procedure[1].

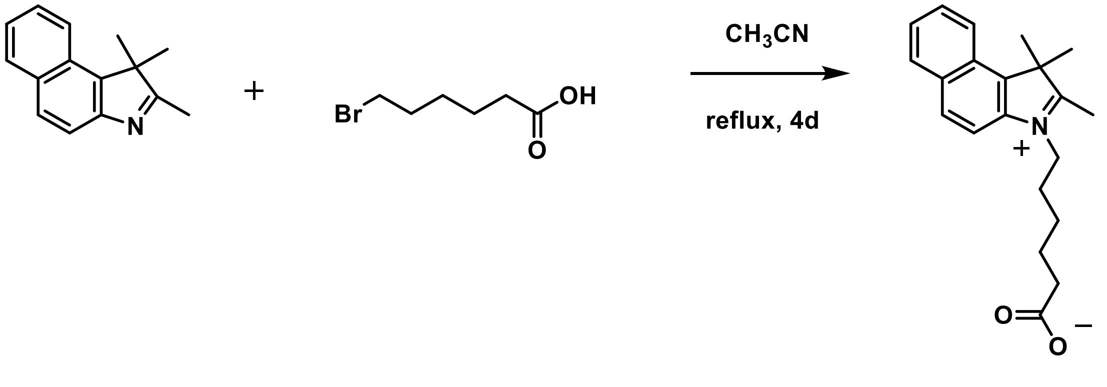

6

Supplementary Figure 5: Synthesis of compound **6**.

6-((E)-2-((2E,4E)-5-(3-(hex-5-yn-1-yl)-1,1-dimethyl-1H-benzo[e]indol-3-ium-2-yl)penta-2,4-dien-1-ylidene)-1,1-dimethyl-1,2-dihydro-3H-benzo[e]indol-3-yl)hexanoate (7) was synthesized adapting a reported procedure.[1]

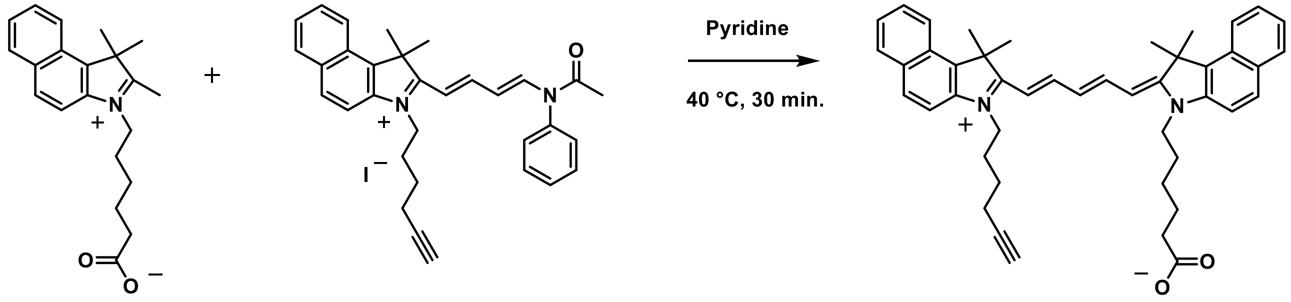

6 5 7

Supplementary Figure 6: Synthesis of compound **7**.

In a 10 mL round bottom flask dried with a heat gun under a flow of Argon, to a solution of the compound **6** (0.110 g, 0.34 mmol) in Pyridine (3 mL) was added the compound **5** (0.157 g, 0.34 mmol) and the reaction mixture was heated at 40°C for 30 min. After cooling down to room temperature, the reaction mixture was concentrated in vacuo and the residue was purified by silica gel chromatography eluting with Dichloromethane/Methanol (99/1 – 90/10) to give the compound **7** as blue solid (0.150 g, yield 68 %). R_f = 0.40 (SiO_2, dichloromethane/Methanol = 90/10).

^{1}H-NMR (400 MHz, $CDCl_3$, 25°C) δ = 8.14 (2H, t, *J* = 7.2 Hz, ar. C*H*), 8.05 (1H, q, *J* = 13.2 Hz, ar. C*H*), 7.96-7.93 (5H, m, ar. C*H*), 7.68-7.61 (2H, m, ar. C*H*), 7.53-7.46 (2H, m, ar. C*H*), 7.40 (1H, d, *J* = 8.8 Hz, -CHC*H*-), 7.34 (1H, d, *J* = 8.8 Hz, -CHC*H*-), 7.09 (1H, t, *J* = 13.2 Hz, -CHC*H*-), 6.73 (1H, d, *J* = 14 Hz, -CHC*H*-), 6.47 (1H, d, *J* = 13.2 Hz, -CHC*H*-), 4.31 (2H, t, *J* = 7.2 Hz, -NCH_2-), 4.18 (2H, t, *J* = 7.6 Hz, -NCH_2-), 2.57 (2H, t, *J* = 6.8 Hz, -CH_2COO$^-$), 2.38-2.34 (2H, m, CHCCH_2-), 2.08-2.06 (2H, m, -NCH_2CH_2-), 2.05 (6H, s, -CH_3), 2.03 (6H, s, -CH_3), 2.02-2.01 (1H, m, -CC*H*), 1.95-1.88 (2H, m, -NCH_2CH_2-), 1.86-1.78 (4H, m, -NCH_2CH_2CH_2-), 1.68-1.61 (2H, m, -CH_2CH_2COO$^-$).

^{13}C-NMR (100 MHz, $CDCl_3$, 25°C) δ = 176.9, 174.1, 152.6, 152.5, 148.7, 139.2, 139.1, 136.8, 134.0, 131.6, 130.4, 129.8, 128.0, 127.6, 126.1, 124.9, 124.0, 122.2, 110.4, 103.2, 103.0, 83.3, 69.4, 51.2, 44.2, 44.0, 34.0, 27.7, 27.1, 26.4, 26.1, 25.3, 24.3, 18.0.

ESI-MS m/z calculated for $C_{45}H_{48}N_2O_2^+$ = 648.4; obs. = 649.2 $(M+H)^+$.

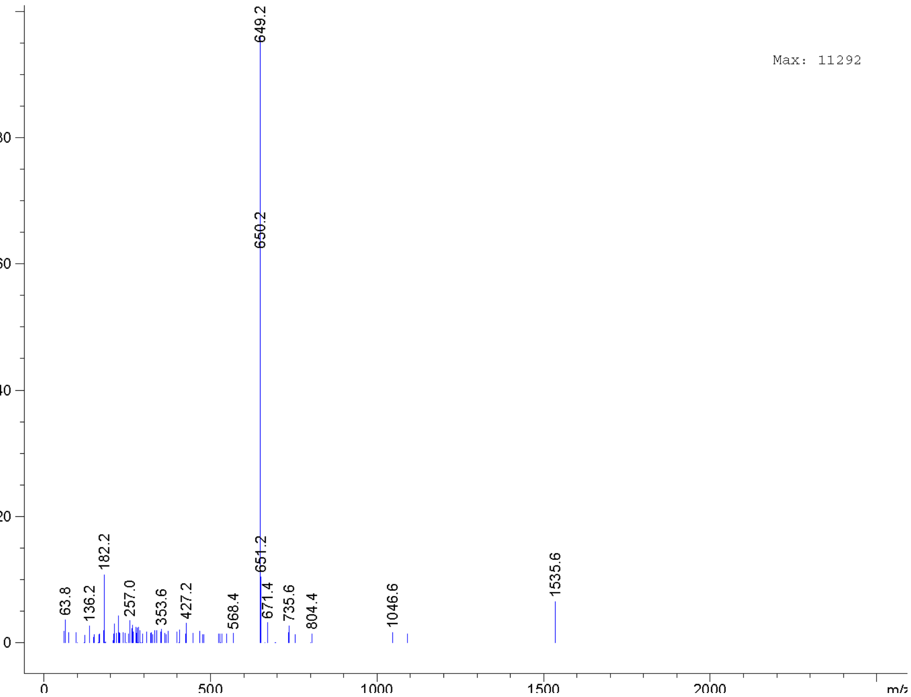

Supplementary Figure 7: ESI-MS spectra of compound **7**.

6-((E)-2-((2E,4E)-5-(3-(4-(1-((1-(tert-butoxycarbonyl)piperidin-3-yl)methyl)-1H-1,2,3-triazol-4-yl)butyl)-1,1-dimethyl-1H-3l4-benzo[e]indol-2-yl)penta-2,4-dien-1-ylidene)-1,1-dimethyl-1,2-dihydro-3H-benzo[e]indol-3-yl)hexanoate (**9**) was synthesized adapting a reported procedure.[2]

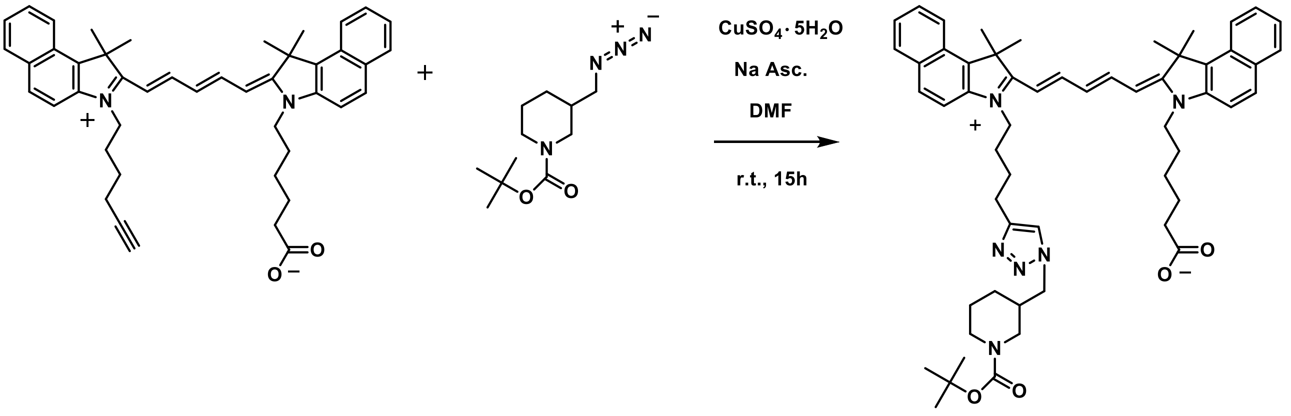

7 **8** **9**

Supplementary Figure 8: Synthesis of compound **9**.

In a 25 mL round bottom flask dried with a heat gun under a flow of argon, tert-butyl 3-(azidomethyl)piperidine-1-carboxylate (**8**, 0.018 g, 0.14 mmol) and the compound **7** (0.110 g, 0.17 mmol) were solubilized with N,N-dimethylformamide (DMF, 8 mL). Copper (II) sulfate pentahydrate ($CuSO_4$ $5H_2O$, 0.035 g, 0.14 mmol) and sodium ascorbate (Na Asc., 0.039 g, 0.20 mmol) were solubilized with 2 mL of water and added to the previous solution, and the reaction mixture was stirred at room temperature overnight. The reaction mixture was poured into 150 mL of diethyl ether; the blue precipitate was then dissolved in 50 mL of dichloromethane and washed three times with lithium chloride solution (1 M in water). The combined organic phases were dried over sodium sulfate and concentrated in vacuo. The residue was dissolved in 5 mL of methanol and poured in mL of diethyl ether; the precipitate was filtered to give the compound **9** as a blue solid (0.070 g, yield 56 %). R_f = 0.56 (silica, chloroform/methanol = 90/10).

^{1}H-NMR (400 MHz, CD_3OD, 25°C) δ = 7.84-7.68 (4H, m, ar. C*H*), 7.63-7.55 (4H, m, ar. C*H*), 7.27-7.23 (2H, m, ar. C*H*), 7.12-7.08 (2H, m, ar. C*H*), 6.94 (1H, s, -NNC*H*-), 7.04-6.98 (2H, m, C*H*), 6.71-6.54 (1H, m, C*H*), 6.15-5.97 (2H, m, C*H*), 3.96-3.78 (4H, m, -NCH_2-), 3.47-3.35 (2H, m, -

NNCH_2-), 3.28-3.03 (4H, m, -COONCH_2-), 2.62 (6H, s, -NCCCH_3), 2.55 (6H, s, -NCCCH_3), 2.48-2.37 (4H, m, -NNCH_2- and -CH_2COO$^-$ overlapped), 1.80-1.22 (15H, m, -CONCH$_2$CH-, -NCH$_2$CH_2-, -NCH$_2$CH$_2$CH_2-, -COONCH$_2$CH_2- and -COONCH$_2$CH$_2$CH_2- overlapped), 1.06 (9H, s, -OCCH_3).

ESI-MS m/z calculated for $C_{56}H_{68}N_6O_4^+$ = 888.5; obs. = 889.4 $(M+H)^+$.

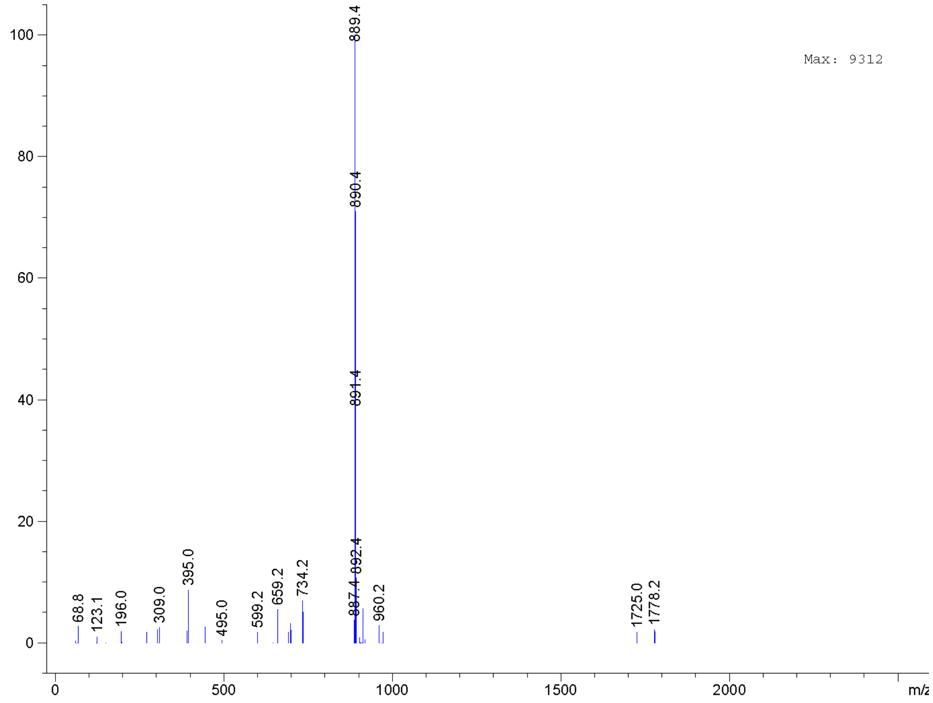

Supplementary Figure 9: ESI-MS spectra of compound **9**.

3-(4-(1-((1-(tert-butoxycarbonyl)piperidin-3-yl)methyl)-1H-1,2,3-triazol-4-yl)butyl)-2-((1E,3E,5E)-5-(1,1-dimethyl-3-(6-oxo-6-((3-(triethoxysilyl)propyl)amino)hexyl)-1,3-dihydro-2H-benzo[e]indol-2-ylidene)penta-1,3-dien-1-yl)-1,1-dimethyl-1H-benzo[e]indol-3-ium (**C5**) was synthesized adapting a reported procedure.[1]

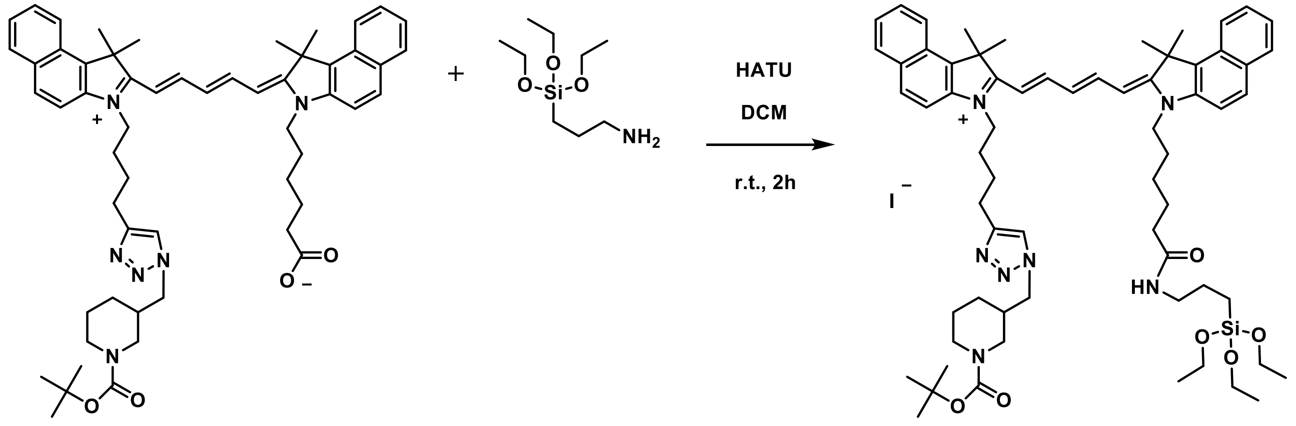

9 **10** **C5**

Supplementary Figure 10: Synthesis of compound **C5**.

In a 25 mL round bottom flask dried with a heat gun under a flow of argon, to a solution of the compound **9** (0.066 g, 74 µmol) in dichloromethane (DCM, 7 mL) was added 1-[bis(dimethylamino)methylene]-1H-1,2,3-triazolo[4,5-b]pyridinium-3-oxid-hexafluoro phosphate (HATU, 0.034 g, 89 µmol), (3-aminopropyl)triethoxysilane (**10**, 0.021 mL, 89 µmol) and N,N-diisopropylethylamine (0.026 mL, 148 µmol) and the reaction mixture was stirred at room temperature for 2h. The reaction mixture was concentrated in vacuo and the residue was purified by silica gel chromatography eluting with dichloromethane/methanol (95/5-80/20) to give the compound **C5** as a blue solid (0.068 g, yield 75%). R_f = 0.73 (SiO_2, dichloromethane/methanol = 90/10).

^{1}H-NMR (400 MHz, CD_3OD, 25°C) δ = 8.38-8.30 (2H, m, ar. C*H*), 8.26 (1H, d, *J* = 4.4 Hz, ar. C*H*), 8.24 (1H, d, *J* = 4.8 Hz, ar. C*H*), 8.04-7.98 (4H, m, ar. C*H*), 7.76 (1H, s, -NNC*H*-), 7.67-7.63 (2H, m, ar. C*H*), 7.61 (1H, d, *J* = 4.4 Hz, ar. C*H*), 7.58 (1H, d, *J* = 4.4 Hz, ar. C*H*), 7.52-7.48 (2H, m, C*H*), 6.68 (1H, t, *J* = 12.4 Hz, C*H*), 6.33 (2H, t, *J* = 13.2 Hz, C*H*), 4.29-4.22 (4H, m, -NCH_2-), 3.79 (6H, q, *J* = 7.2 Hz, -OCH_2-), 3.64 (1H, s, -CON*H*-), 3.10 (2H, t, *J* = 7.2 Hz, -NHCH_2-), 2.99-2.81 (7H, m, -NNCH_2-, -COONCH_2- and –NNCC*H*- overlapped), 2.22 (2H, t, *J* = 7.2 Hz, -CH_2CONH-), 2.04 (6H, s, -NCCCH_3), 2.02 (6H, s, -NCCCH_3), 1.93-1.87 (4H, m, -NCH$_2$CH_2-), 1.78-1.48 (12H, m, -COONCH$_2$CH_2-, -COONCH$_2$CH$_2$CH_2-, -NCH$_2$CH$_2$CH_2-, -NHCOCH$_2$CH_2- and -SiCH$_2$CH_2- overlapped), 1.34 (9H, s, -OCCH_3), 1.30-1.27 (1H, m, -CONCH$_2$C*H*-), 1.18 (9H, t, *J* = 6.8 Hz, -OCH$_2$CH_3), 0.59-0.55 (2H, m, -SiCH_2-).

ESI-MS m/z calculated for $C_{65}H_{90}N_7O_6Si^+$ 1092.7; obs.: 1092.4.

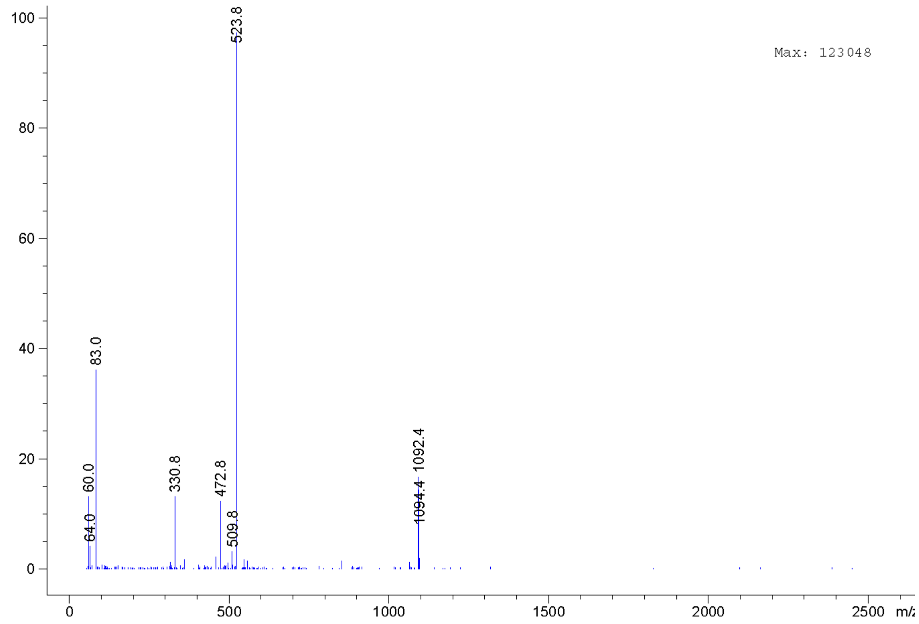

Supplementary Figure 11: ESI-MS spectra of compound **C5**.

2-((E)-2-((E)-2-((4-carboxyphenyl)thio)-3-(2-((E)-1,3,3-trimethylindolin-2-ylidene)ethylidene)cyclohex-1-en-1-yl)vinyl)-1,3,3-trimethyl-3H-indol-1-ium chloride (**12**) was synthesized following a reported procedure.[1]

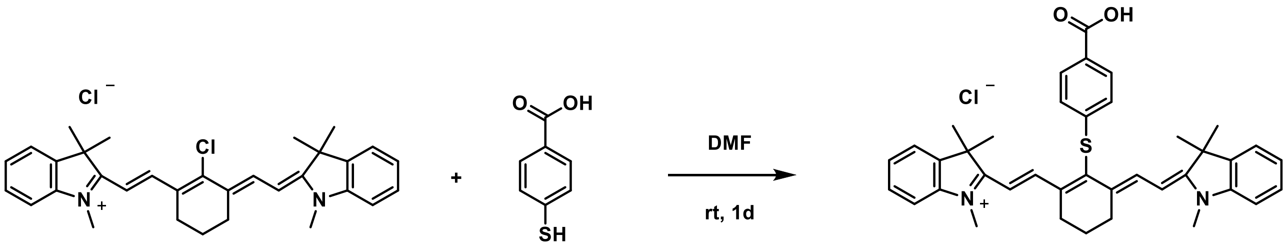

12

Supplementary Figure 12: Synthesis of compound **12**.

1,3,3-trimethyl-2-((E)-2-((E)-2-((4-((3-(triethoxysilyl)propyl)carbamoyl)phenyl)thio)-3-(2-((E)-1,3,3-trimethylindolin-2-ylidene)ethylidene)cyclohex-1-en-1-yl)vinyl)-3H-indol-1-ium chloride (**13**) was synthesized adapting a reported procedure.[2]

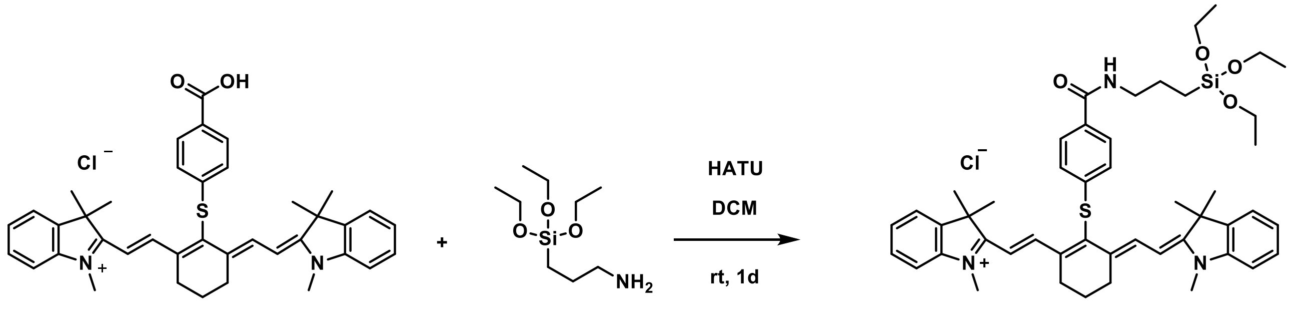

12 **APTES** **C7**

Supplementary Figure 13: Synthesis of compound **C7**.

In a 25 mL round bottom flask dried with a heat gun under a flow of argon, to a solution of the compound **12** (0.131 g, 0.20 mmol) in dichloromethane (7 mL) were added 1-[bis(dimethylamino)methylene]-1H-1,2,3-triazolo[4,5-b]pyridinium-3-oxid-hexafluoro phosphate (HATU, 0.091 g, 0.24 mmol), (3-aminopropyl)triethoxysilane (APTES, 0.056 mL, 0.24 mmol) and N,N-diisopropylethylamine (0.070 mL, 0.40 mmol) and the reaction mixture was stirred at room temperature for 24h. The reaction mixture was concentrated in vacuo and the residue was purified by silica gel chromatography eluting with dichloromethane/methanol (95/5-75/25) to give the compound **C7** as a green solid (0.102 g, yield 61 %). R_f = 0.54 (silica, dichloromethane/methanol = 85/15).

^{1}H-NMR [400 MHz, $(CD_3)_2SO$, 25°C)] δ = 8.55 (2H, d, *J* = 14, C*H*), 8.32 (1H, t, *J* = 5.6 Hz, -CON*H*-), 7.76 (2H, d, *J* = 8.4 Hz, ar. C*H*), 7.51 (2H, d, *J* = 7.6 Hz, ar. C*H*), 7.44-7.39 (4H, m, ar. C*H*), 7.32 (2H, d, *J* = 8.4 Hz, ar. C*H*), 7.26-7.21 (2H, m, ar. C*H*), 6.31 (2H, d, *J* = 14 Hz, C*H*), 3.68 (6H, q, *J* = 7.2 Hz, -OCH_2-), 3.64 (6H, s, -NCH_3-), 3.18-3.13 (2H, m, -NHCH_2-), 2.80-2.77 (4H, m, -SCCCH_2-), 1.98-1.91 (2H, m, -SCCCH$_2$CH_2-), 1.54-1.46 (2H, m, -NHCH$_2$CH_2-), 1.41 (12H, s, -CCH_3), 1.08 (9H, t, *J* = 6.8 Hz, -OCH$_2$CH_3), 0.54-0.50 (2H, m, -SiCH_2-).

ESI-MS m/z calculated for $C_{48}H_{62}N_3O_4SSi^+$ = 804.4; obs. = 804.2.

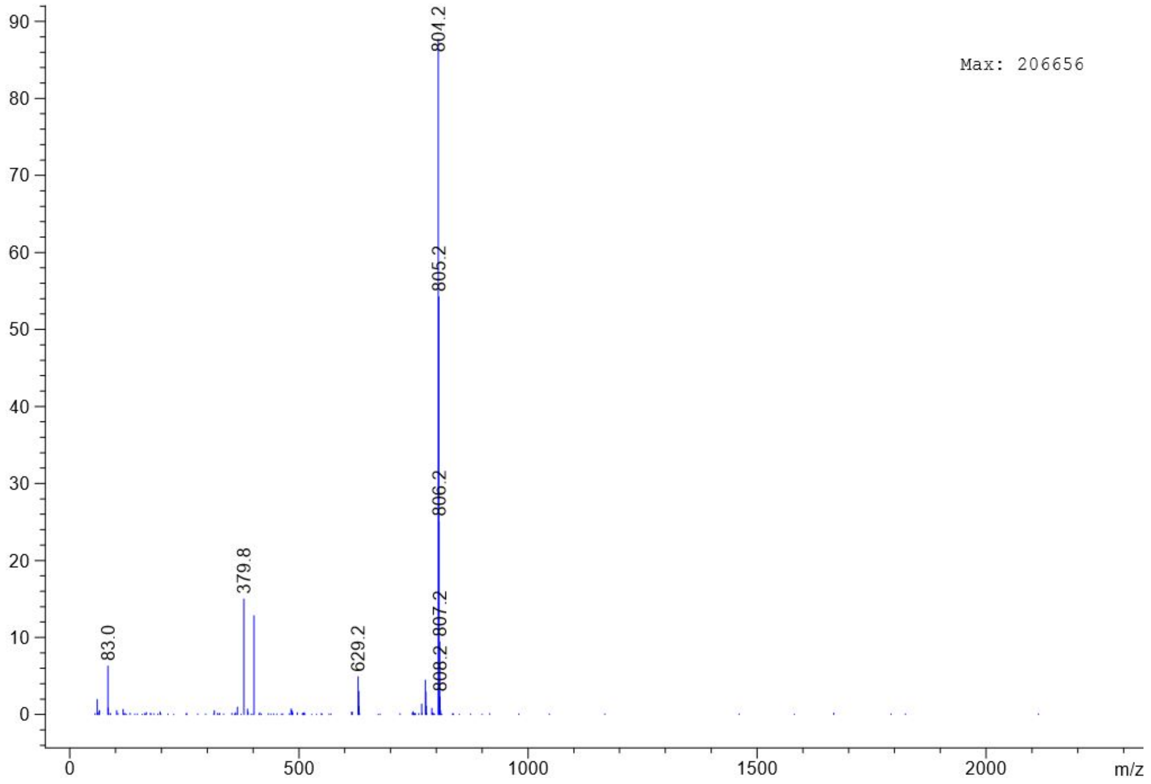

Supplementary Figure 14: ESI-MS spectra of compound **C7**.

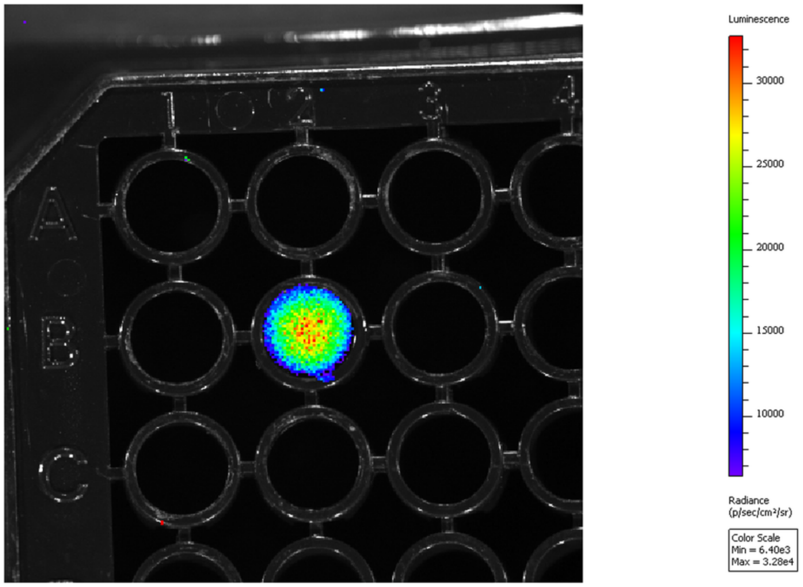

Supplementary Figure 15: Optical image of PluS NPs and 32P- ATP acquired in bioluminescence modality in non-fluorescent 96-multwell plate.

References

1. Biffi S, Petrizza L, Rampazzo E, et al. Multiple dye-doped NIR-emitting silica nanoparticles for both flow cytometry and in vivo imaging. *RSC Adv.* 2014;4:18278-18285.

2. Petrizza L, Collot M, Richert L, et al. Dye-doped silica nanoparticle probes for fluorescence lifetime imaging of reductive environments in living cells. *RSC Adv.* 2016;6:104164-104172.
